# Epidemiology of Viral Hepatitis and Liver Diseases in Pakistan

**DOI:** 10.5005/jp-journals-10018-1129

**Published:** 2015-01-06

**Authors:** Amna Subhan Butt

**Affiliations:** Department of Medicine, Aga Khan University Hospital, Karachi, Pakistan

**Keywords:** Hepatitis in Pakistan, Liver diseases in Pakistan, Hepatitis B and C in Pakistan.

## Abstract

Pakistan is among ten most populous countries in world. All sorts of hepatitis are highly prevalent in Pakistan. Due to lack of proper mechanisms to handle sewerage, disinfection and lack of clean water supply across the country, hepatitis A and E are endemic in Pakistan. Moreover, Pakistan ranked in intermediate prevalence zone for hepatitis B and C. Also, prevailing socioeconomic conditions, political statuses and unstable healthcare delivery system are not suitable for tackling further progression of hepatitis viruses and its complications. The notable factors related to epidemiology of different hepatitis viruses have been described in this communication.

**How to cite this article:**

Butt AS. Epidemiology of Viral Hepatitis and Liver Diseases in Pakistan. Euroasian J Hepato-Gastroenterol 2015;5(1):43-48.

## INTRODUCTION

Affecting millions of people worldwide, viral hepatitis is a global health problem. The prevalence, most common transmission pathways and pathogenicity of hepatitis vary across the world as well as within countries. Transmitting via exposure to contaminated blood, hepatitis B virus (HBV), hepatitis C virus (HCV) and hepatitis D virus (HDV) leads to chronic hepatitis in majority of cases.^1^ Hepatitis A (HAV) and E (HEV) transmitted primarily via fecal-oral route and mostly resolved after causing acute hepatitis.^1^ Pakistan ranked as a low to middle income country among 22 member states of World health organization (WHO) eastern mediterranean region (EMR). Moreover, with the population of 176.7 million, Pakistan is among 10 most populated countries in the world. Not only in EMRO region but globally as well, Egypt and Pakistan are among the counties where hepatitis are highly prevalent.^1^ Furthermore, there is additional strain on existing health systems due to major social and political upheavals, active or proxy wars in different parts of country, internal displacements of large proportions of persons per population and lack of access to effective healthcare services. Hence, leading to ideal conditions for the spread of hepatitis, especially those spread by contaminated water and lack of sanitation services.^2^

## HEPATITIS A AND E INFECTION IN PAKISTAN

Most of acute hepatitis A and E infections remain clinically indistinguishable from other causes of acute viral hepatitis. According to WHO estimates, every year 1.4 million new cases of HAV and 20 million of HEV are reported globally. Approximately, 100,000 people/year and 60,000 people/year die due to acute HAV and HEV infection respectively. However, the attributed mortality rate is even much higher among pregnant women.^1,2^ Unfortunately, there are very few community-based studies and most of the reported evidence regarding prevalence of HAV and HEV in Pakistan are from hospital/clinic-based studies. Hence, there is variation in the reported statistics. However, it has been that HAV and HEV are endemic in Pakistan.

Hepatitis A accounts for 50 to 60% of acute viral hepatitis in children of Pakistan. Attributed to early exposure, almost 96% of individuals are found to be exposed (HAV IgG reactive) to HAV by the age of 5 years.^3,4^ On the other hand, HAV leads to acute hepatitis in 3.5 to 4% of adults and approximately 98 to 100% adults are exposed to HAV at adulthood.^5^ Acute HAV infection is usually subclinical or resolve without complications. However, in a series of 2,735 confirmed cases of acute HAV, reported from 1991 to 1998 from a Tertiary Care Hospital of Pakistan, 232 children required hospitalization and of these 36.7% died.^6^ These findings supports need of improved sanitation, provision of clean water and early vaccination of children for HAV in countries like Pakistan. These measures will be helpful in reducing morbidity and mortality associated with HAV (Table 1).

Being a most common cause of acute hepatitis in adults, HEV is responsible for acute hepatitis in 20 to 22% among adults and 2.4% among children.^3^ Past exposure to HEV has been reported in 1.4 to 19.4% children and 16 to 20% of healthy adults.^3,4,7^ Due to the fecal contamination of water, mini outbreaks have been reported mostly during summers, rain and floods with attack rate ranging 10 to 20 (Table 2).^3,8,9^ The overall mortality rate due to acute hepatitis E ranges 0.4 to 4.0%; however, mortality rate gets much higher in the presence of pregnancy (16-33%)^10,11^ and incase of acute liver failure with HEV in the presence of chronic liver disease. So far, HEV genotype 1 has been reported as the dominant genotype in Pakistan with isolates Sar-55 (87-Pakistan-A), Abb-2B (88-Pakistan-2B) and 87-Pakistan-B.^12^ Contamination of sewerage with water resources has a major role in HEV spread in Pakistan. In a recent study, 86 raw sewerage samples were collected from different outlets of Islamabad (capital of Pakistan) and Rawalpindi (neighboring city to Islamabad). Out of these 19(44.7%) samples from Rawalpindi and 16 (41.02%) from Islamabad were HEV positive. All of HEV positive samples were from the highly congested areas of two cities.^13^ These findings clearly suggest that the lack of proper mechanism to handle sewerage and disinfection is a leading factor for spread of HEV.

## HEPATITIS B, C AND D IN PAKISTAN

Hepatitis B and C are responsible for >75% of cirrhosis and hepatocellular carcinoma (HCC) in WHO EMRO region.^2^ Variable prevalence estimates has been reported in various studies from Pakistan which is most probably due to differences in study design, sample size, study settings and exposure to various risk factors. Most of the studies have reported HCV prevalence among children between 0.4 and 1.4% except one study from Myo Hospital, Lahore, where out of 538 healthy children, 4.0% were found to have reactive anti-HCV antibody.^3,14-16^ The prevalence of HBV ranged between 1.9 and 3.6% among healthy children (Table 3).^3,4^

Among adults several cross-sectional studies have been conducted that revealed prevalence of HBV 2.2 to 11.9% in healthy individuals. Relatively lower HBV prevalence was found among individuals who were screened prior to employment in armed forces (3-7.3%) and healthy blood donors (0.8-5.8%). A wide variability in HBV prevalence estimates has been observed among pregnant women, i.e. 1.6 to 12.0%. Being at risk healthcare workers had higher HBV prevalence that ranged from 2.4 to 20% with the dentists (17%) and housekeeping (20%) staff being the most affected one. The estimated HBV prevalence was alarming among high-risk individuals like commercial sex workers (11.6%), individuals with thalassemia/hemophilia (4-8.4%), those who were on regular hemodialysis (6.9-12.4%), prisoners (5.9%) and drug addicts (22.8%).^3,15^ The most common HBV genotype in Pakistan is genotype D (65-96.2%) followed by genotype C.^3,17^

**Table 1:** **Table 1:** HeDatitis A in Pakistan^3,4,30^

		*Anti-HAV IgG (%)*		*Anti-HAVIgM (%)*	
Healthy children		82-100		—	
Children with sub-clinical heDatitis		93.2		—	
Children with acute viral heDatitis		—		52	
Healthy adults		92.0, 96.6		—	
Adult Datients with acute viral hepatitis		—		3.5-4.0	

**Table 2:** **Table 2:** Hepatitis E in Pakistan^3,4,7,30-32^

		*Anti-HEV IgG/ total+ (%)*		*Anti-HEV* *IgM+ (%)* or n	
Healthy children		18.3, 19.4		—	
Children from low socioeconomic urban communities		1.4, 14.4		2.4	
Healthy adults		16.0, 20		—	
Hospitalized patients with jaundice/acute hepatitis		7-22.0		20.2	
PAF bases Karachi, outbreak of acute hepatitis		—		204 confirmed cases	
G-10 Islamabad, outbreak of HEV in general public		—		3827, 10.0 AR	
Lahore Garrison, Outbreak of HEV in army people		—		283 confirmed	
Military Unit Abbottabad, outbreak of hepatitis E in military unit (n = 109)				95.0	
Hospital acquired outbreak, Karachi (N = 113)		—		AR 15.9	
Pregnant women with jaundice/ acute hepatitis		57.0, 61		66.9	
HEV superinfection in CLD		17.5		—	

The overall prevalence of HCV among volunteer blood donors and healthy adults ranged between 0.2 and 6.5% and 2.1 to 13.5% respectively. As compared to HBV, the HCV prevalence was found much higher among pregnant women (3.2-18.2%). Those who had recurrent exposure to blood or blood products like those who have thalassemia/ hemophilia (25-56.8%) and those who are hemodialysis dependent (20-24.7%) were found to have highest HCV prevalence which is very alarming.^3,15,16^ Owing to high-risk behavior prisoners were also found to have high HCV prevalence, i.e. 15.2%.^18^ Majority of patients (>80%) are infected by HCV genotype 3. Other relatively common HCV genotypes are 2 and 1.^3,16^

**Table 3:** **Table 3:** Hepatits B virus and HCV in Pakistan^3,15,16,24^

		*HBsAg+(%)*		*Anti-HCV+*	
Healthy children		1.9-3.6		0.4-1.4	
Healthy adult recruits in		3-7.3		2.2-5.2	
armed forces					
Blood donors		0.8-5.8		0.2-6.5	
Healthy adults/		2.2-11.9		2.1-13.5	
general population					
Pregnant women		1.6-12		3.2-18.2	
Spouses of index patients		—		4.3-5.1	
Healthcare workers		2.4-20		4-10	
Commercial sex workers		11.6		12.3	
Thalassemia/hemophilia		4-8.4		25-56.8	
Dialysis		6.9-12.4		20-24.7	
Chronic liver disease		10-46.67		40-86.0	
Hepatocellular carcinoma		25.35		57.99	

Considering the significant burden of HBV and HCV differences in reported estimates from different areas of Pakistan, first national survey was conducted during 2007 to 2008, to estimate prevalence of hepatitis B and C in general population of Pakistan.^19^ A total of 47,043 individuals were screened. The overall prevalences of HBV and HCV were 2.5 and 4.8% respectively, reflected a combined infection rate of 7.6% in the general population. Increasing age and being married, exposure to therapeutic injections, hospitalization, and shave by barbers were the factors associated with higher risk of acquiring HBV and HCV. Among all four provinces, the highest HCV prevalence was found in Punjab (6.7%) and Sindh (5.0%) which was even much higher (12-13%) in certain cities of both provinces. The highest prevalence of HCV was found in Balochistan (4.3%) followed by Sindh (2.5%).

Hepatitis D is another challenge for healthcare providers in Pakistan. Hepatitis D virus could present as coinfection or superinfection with HBV. In an earlier study of 408 patients with hepatitis B-related chronic liver disease, 44 and 1.4% patients had HDV superinfection and coinfection respectively.^20^ Based upon presence of anti-HDV antibody, the prevalence of HDV was found 16.6 to 35.2% in subsequent studies.^21,22^ Mumtaz et al compared BV/HDV coinfection with HBV mono-infection. In comparison to HBV mono-infection, HBV/ HDV coinfection was associated with higher ALT levels, suppressed HBV DNA levels, HBeAg negative disease and more aggressive liver disease including cirrhosis.^22^

According to WHO estimates up to 75% of therapeutic injections received in southeast Asia are unsafe. The scenario applies well to Pakistan where syringes are reused and sterility of injections is often not maintained, hence, play a major role in spread of HBV/HCV. In Pakistan, approximately 1.5 million units of blood products are transfused every year. Unfortunately, the standardized practices in provision of safe blood products are not followed by all blood banks, leading to spread of hepatitis B, C and D. Exposure to contaminated instruments or blood during minor/major surgeries, sharing sharps like razors, males getting their shave from communal barber, prior hospitalizations, injections drug use, body piercing, perinatal transmission, high risk sexual behavior are the other factors identified attributing to the spread of hepatitis B, C and D.^15,16,19,23^

## ACUTE HEPATITIS, CHRONIC LIVER DISEASE AND HEPATOCELLULAR CARCINOMA IN PAKISTAN

In case series reported from one of the largest government sector Tertiary Care Hospital of Karachi, Pakistan, the authors analyzed 5,193 cases fulfilling all criteria of viral hepatitis and admitted during 1987 to 2007.^5^ Out of these, 6.7% had acute hepatitis, 44.1°% had chronic hepatitis, 27.5% had cirrhosis and 20.8% were HBV/HCV carriers. Hepatitis C was found to be the most common (55.8%) viral hepatitis, associated with chronic hepatitis and cirrhosis followed by hepatitis B (32.6%). Hepatitis B was seen a decade earlier than hepatitis C. Over decades a rising trend in HCV and declining evidence of hepatitis B was observed which could be due to increased awareness and detection of hepatitis C and efforts for HBV vaccination in Pakistan. Likewise, data from other studies revealed 40 to 86% and 10 to 46% cases of chronic liver disease are attributed to hepatitis C and B respectively.^3,15,16^

Hepatocellular carcinoma is a leading cause of cancer-related mortality. Based upon data from selected, local cancer registries from Pakistan, HCC was found to be the third most common cancer in men (age standardized rate 12.3/100,000) and the seventh most common cancer in women (ASR 3.1/100,000).^24^ Unfortunately, most of the studies available from Pakistan are hospital-based case series with small sample size; hence true prevalence and incidence rate of HCC could not be ascertained. Recently, Butt et al^24^ have reported an aggregated analysis of 3,319 cases from 29 studies. The HCC was attributed to HCV in 57.99%, HBV in 25.35%, HBV/HCV coinfection in 5.26% cases. Additionally, HBV/HDV and HBV/HDV/HCV coinfections were found in approximately 2% cases. Beside a consistent rising trend in number of HCC cases reported, a clear shift was observed in etiological factors leading to HCC. From the 1970s till the mid 1990s, hepatitis B was the most common etiological factor for HCC which was replaced by HCV afterwards. This is might due to the rising incidence of DM and metabolic syndrome that increasing number of viral marker negative HCC has been reported over the period of time. In the largest study reported from Pakistan, out of 645 HCC cases, 15.3% were viral marker negative.^25^ Unfortunately, majority (62.8%) of patients had advanced HCC on presentation, diagnosed when symptomatic. Moreover, HBV-HCC and viral marker negative HCCs were found to be more advanced and aggressive at the time of presentation. Compliance with surveillance was found poor in case of nonviral HCC.

## EFFORTS TO CONTROL HEPATITIS B AND C IN PAKISTAN

1. *Establishment of a viral hepatitis surveillance system:* In 2005, Ministry of Health (MOH) has launched a National Program for Hepatitis Prevention and Control (NPHPC) which was mainly dealing with HCV and had many limitations. In collaboration with CDC’s Division of Viral Hepatitis, Pakistan Field Epidemiology and Laboratory Training Program (FELTP) established a hepatitis sentinel surveillance system in five large public hospitals in four provinces and Islamabad capital territory in 2009. Field epidemiology and laboratory training program is housed at National Institute of Health in Islamabad.^26^ From June 2013 to December 2014, out of 7,387 suspected cases, 3,008 had viral hepatitis. The newly reported HCV cases were 65%. Hepatitis E, B was found in 12, 18, 31.4 and 2.3% cases respectively. Majority of HAV or HEV-infected patients were 6 to 30 years of age. While those who had hepatitis B or C were comparatively older.^26,27^
2. *Prime Minister’s program for the prevention and control of hepatitis viral infections:* Considering heavy burden and huge cost, the Prime Minister’s Program for the prevention and control of hepatitis viral infections was launched for 5 years from 2005 to 2010, to support treatment of hepatitis B and C for nonaffording patients. A total of 7,752 patients were treated at the 12 sites for hepatitis C. Only 45.4% patients completed 6 months of interferon and ribavirin therapy and end of treatment response was available in 49% cases. Approximately, 67% had EOTR and 33% were non-responders. Data for hepatitis B were collected from eight sites. A total of 454 cases received treatment and 85 (18.72%) fulfilled the criteria for treatment. Treatment was completed by nine (10.58%) cases, with three (3.52%) cases showing Hepatitis B ‘e’ antigen clearance and anti-HBe production.^28^ However, proper planning and accountability lack in this program. Inadequate clinical follow-up and inadequate documentation of serological/biochemical tests were the major drawbacks, hence, leading to inadequate assessment of treatment response, failure to complete treatment, resulting in wastage of huge human and financial resources.
3. *Immunization for HBV:* The HBV prevalence among children <5 years of age has been found quite high which is probably due to vertical transmission. A tetravalent vaccine (with DPT) HBV vaccine was incorporated in Pakistan’s EPI in 2001. It was later replaced with the pentavalent vaccine (DPT, HBV, Hib) given at 6, 10 and 14 weeks without a birth dose. In a recent study mothers and their children aged 6 to 36 months were tested for HBV to assess the risk of HBV transmission in infants born to HBV positive mothers in Pakistan.^29^ Children born to HBsAg positive mothers had higher prevalence of HBV (14.6%) as compared to HBsAg negative mothers (2.1%). Moreover, HBV transmission rate to infants was found 5.4% by 12 months of age when mother was HBeAg positive. Despite provision of facility for HBV vaccination in Prime Minister Program for adults, heathcare providers, the vaccine coverage is low. This may be due to lack of awareness at community level or inability to access the facility.
4. *Other measures:* To increase awareness, certain measures have been taken at government and community level. To improve awareness and to establish partnerships, government has funded other viral hepatitis public awareness campaigns since January 2011 and held events for World Hepatitis Day 2012 and later. Many NGOs, GI and liver disease societies conduct seminars, mass campaigns to educate community and healthcare providers. Antiviral therapies for HBV (interferon alpha, pegylated interferon, lamivudine and entecavir) and HCV (interferon alpha, pegylated interferon and ribavirin) are on the national essential medicines list or subsidized by the government. A standard definition for hepatitis has been defined. However, beside recently established FELTP, there is no system for routine surveillance and reporting of outbreaks, mortalities due to hepatitis, liver disease and HCC.

## CONCLUSION

Burden of hepatitis and its complications are a huge challenge for government and healthcare providers in Pakistan. Majority of patients remain asymptomatic, leading to silent epidemic. Measures for prevention and control of hepatitis can, therefore, make a significant contribution in reducing the disease burden and saving lives. A high proportion of patients approach private hospitals for treatment and bear heavy cost for their treatment. Public health services system constitutes the only source of care available for the nonaffording patients. Prime Minister’s program for the prevention and control of hepatitis viral infections is a unique program in the region. However, most of the efforts and resources were utilized in treatment rather than prevention and the program is victimized by poor planning and lack of quality control measures. The cost to treat patients with chronic HBV or HCV infection far outweighs the cost of implementing prevention programs. A comprehensive strategy is urgently needed to prevent transmission of these blood-borne pathogens. There is an urgent need to implement National Blood Policy regarding provision of safe blood products, to organized transfusion network, to establish surveillance system to report blood born infections, standardization and regulation of appropriate blood screening procedures. Public awareness campaigns are needed to educate people and even healthcare providers about factors associated with spread of hepatitis, especially to avoid reuse of therapeutic injections, sharps like razors, etc. Measures are needed to rehabilitate IDUs, implementation of steps like needle exchange program and vaccination for HBV at least. Hepatitis B virus vaccination should be provided at birth and measures are needed to improve HBV vaccine coverage. Strategies to improve safe, clean water supply and to improve sewerage system are imperative in prevention of HAV, HEV. Hence, it is a long journey and huge efforts are needed to reduce burden of hepatitis and its complication in Pakistan.
